# Analysing the six-year malaria trends at Metehara Health Centre in Central Ethiopia: the impact of resurgence on the 2030 elimination goals

**DOI:** 10.1186/s12936-024-04854-w

**Published:** 2024-01-23

**Authors:** Aynalem Mandefro, Geletta Tadele, Bacha Mekonen, Lemu Golassa

**Affiliations:** 1 Akililu Lemma Institute of Pathobiology, Addis Ababa University, Addis Ababa, Ethiopia; 2 College of Medicine and Health Science, Wollega University, Nekemte, Ethiopia; 3 Malaria and NTDs Research Team, Bacterial, Parasitic, and Zoonotic Diseases Research Directorate, Ethiopian Public Health Institute, Addis Ababa, Ethiopia

**Keywords:** Malaria, Metehara, Retrospective, Trend

## Abstract

**Background:**

Despite Ethiopia’s concerted efforts to eliminate malaria by 2030, the disease continues to pose a significant public health and socioeconomic challenge in the country. The year 2021 witnessed 2.78 million malaria cases and 8041 associated deaths, emphasizing the persistent threat. Monitoring the prevalence trend of malaria is crucial for devising effective control and elimination strategies. This study aims to assess the trend of malaria prevalence at the Metehara Health Centre in the East Shoa Zone, Ethiopia.

**Methods:**

A retrospective study, spanning from February to September 2023, utilized malaria registration laboratory logbooks at Metehara Health Centre to evaluate the prevalence of malaria from 2017/18 to 2022/23. Malaria and related data were collected using a pre-designed data collection sheet. Descriptive statistics were employed for data summarization, presented through graphs and tables.

**Results:**

Out of 59,250 examined blood films, 17.4% confirmed the presence of *Plasmodium* infections. Among the confirmed cases, 74.3%, 23.8%, and 1.84% were attributed to *Plasmodium falciparum*, *Plasmodium vivax*, and mixed infections, respectively. The trend of malaria exhibited a steady decline from 2017/18 to 2021/22, reaching 9.8% prevalence. However, an abrupt increase to 26.5% was observed in 2022/23. Males accounted for a higher proportion (66%) of cases compared to females (34%). The age group 15–24 years experienced the highest malaria incidence at 42%. Notably, malaria cases peaked during autumn (September to November) at 43% and reached the lowest percentage during spring (March to May) at 13%.

**Conclusion:**

Malaria persists as a significant health challenge in and around Metehara, central Ethiopia, predominantly driven by *Plasmodium falciparum*. The five-year declining trend was interrupted by a notable upsurge in 2022/23, indicating a resurgence of malaria in the study area. It is imperative to adopt a reverse strategy to sustain the progress achieved by the national malaria control plan.

## Background

 While substantial strides have been achieved in mitigating the morbidity and mortality associated with malaria, it persists as a formidable global public health challenge. In the year 2021 alone, there were 244 million reported cases and 610,000 deaths worldwide, with Africa bearing the predominant burden, accounting for over 95% of global cases and fatalities [1]. Within Ethiopia, where around 60% of the population resides in malaria-prone areas [2, 3], 2.78 million cases and 8041 deaths were reported in 2021 [1]. The repercussions of malaria extend beyond immediate health concerns, significantly impacting socioeconomic aspects, particularly in rural communities affecting over 80% of the population [4]. In Ethiopia, approximately 70% of reported malaria cases are attributed to *Plasmodium falciparum*, while *Plasmodium vivax* accounts for roughly 30% [3]. The primary mosquito species responsible for transmitting malaria is *Anopheles arabiensis*, with additional contributions from *Anopheles pharoensis*, *Anopheles nili*, and *Anopheles funestus* [2, 3]. Recent reports have also identified *Anopheles stephensi*, an Asian malaria vector [5, 6].

For uncomplicated cases of *Plasmodium falciparum* malaria, artemether-lumefantrine is recommended as the first-line treatment, while chloroquine is used for uncomplicated *Plasmodium vivax* cases [3]. Malaria transmission in Ethiopia is characterized by diverse patterns that are low, unstable, and exhibit geographical and temporal variations [7]. The primary transmission season spans from September to December, following the main rainy season from June to August, with a minor transmission season observed from March to May [8, 9].

The variations in climate factors such as temperature and rainfall are the major causes of malaria’s seasonality [10, 11]. Temperature windows of 15 to 40 °C are ideal for seasonal malaria transmission [12]. Transmission of the disease cannot occur below 15 °C, and temperatures above 40 °C have a negative impact on mosquito population turnover [12, 13]. Variations in rainfall also have an impact on mosquito distribution, survival, and breeding, which in turn affects malaria transmission [12–14]. Weather events such as heavy rainfall create mosquito breeding habitats through flooding, while droughts result in water scarcity, leading to the storage of water in containers that can become breeding sites [15]. In addition, these weather events can displace populations, disrupt healthcare services, and facilitate the rapid spread of malaria.

The World Health Organization has endorsed the Global Technical Strategy (GTS) 2016–2030, advocating for a community-based approach to enhance access to prevention, diagnosis, and treatment with the goal of eliminating malaria by 2030 [16]. Ethiopia has embraced the GTS and developed a strategic plan for malaria elimination, aiming to target districts with an annual parasite index of less than 10 by 2025 and achieve complete elimination by 2030 [2, 3, 17]. Despite a decline in malaria morbidity and mortality in the country, challenges persist due to the development of insecticidal resistance by *An. arabiensis* [18, 19] and *An. stephensi* [20], as well as the emergence of diagnostic failures for *P. falciparum* [21–23]. Ongoing armed conflicts in different parts of Ethiopia, including Northern Ethiopia, Oromia, and Benishangul-Gumuz, coupled with population displacement due to drought, impede efforts to control and eliminate malaria [17, 24–26]. Several risk factors arise in areas affected by conflict, including breakdown of health centres, displacement of large non-immune populations to endemic areas and resettlement of refugees to deteriorated environments that favour vector breeding [24, 27, 28].

Moreover, conflicts can disrupt vector control programmes aimed at reducing mosquito populations through techniques like indoor residual spraying and the distribution of insecticide-treated bed nets [27]. Consequently, these circumstances contribute to the emergence and spread of malaria, hindering access to medical services and diminishing the effectiveness of prevention strategies [29, 30].

It is essential to comprehend the trends, seasonal patterns, and distribution of malaria to guide the Metehara health services and evaluate the progression in malaria control and elimination efforts. Metehara town is situated in Central Rift Valley which is vulnerable to volcanic eruptions, creating stagnant water pools ideal for mosquito breeding after the rainy season [31–33]. The Awash River Basin, Lake Besaka, and agricultural activities like Metehara Sugar factory and Fentale irrigation project increase the risk of malaria transmission [5, 32, 34]. Besides, Metehara acts as a hub between Adama and Awash cities on the Addis-Djibouti highway, attracting a flow of travellers, drivers, and tourists who often stop for rest. This movement of people increases the risk of malaria transmission by facilitating contact between mosquitoes and humans [35]. Outdoor gatherings in hotels, bars, restaurants, and shopping centres in Metehara, particularly during nighttime when mosquitoes are most active, further contribute to the potential for increased transmission of malaria. Trends analysis of malaria prevalence, seasonal pattern and distribution could help in the understanding of disease transmission dynamics. This information is necessary for evidence-based and area-specific interventions. The current understanding of the malaria burden in the Metehara health centre remains largely unexplored. Nevertheless, to guide the town toward malaria elimination, accurate evaluations of the burden, including trends, seasonal patterns, and distribution, are essential. This study specifically endeavors to assess the current prevalence and trend of malaria over the last 6 years in the Metehara Health Centre in the East Shoa Zone of Ethiopia. By addressing this knowledge gap, the study contributes valuable insights for the development of targeted interventions and strategies aimed at the effective control and elimination of malaria in the study area.

## Methods

### Study area and study population

**Fig. 1:**
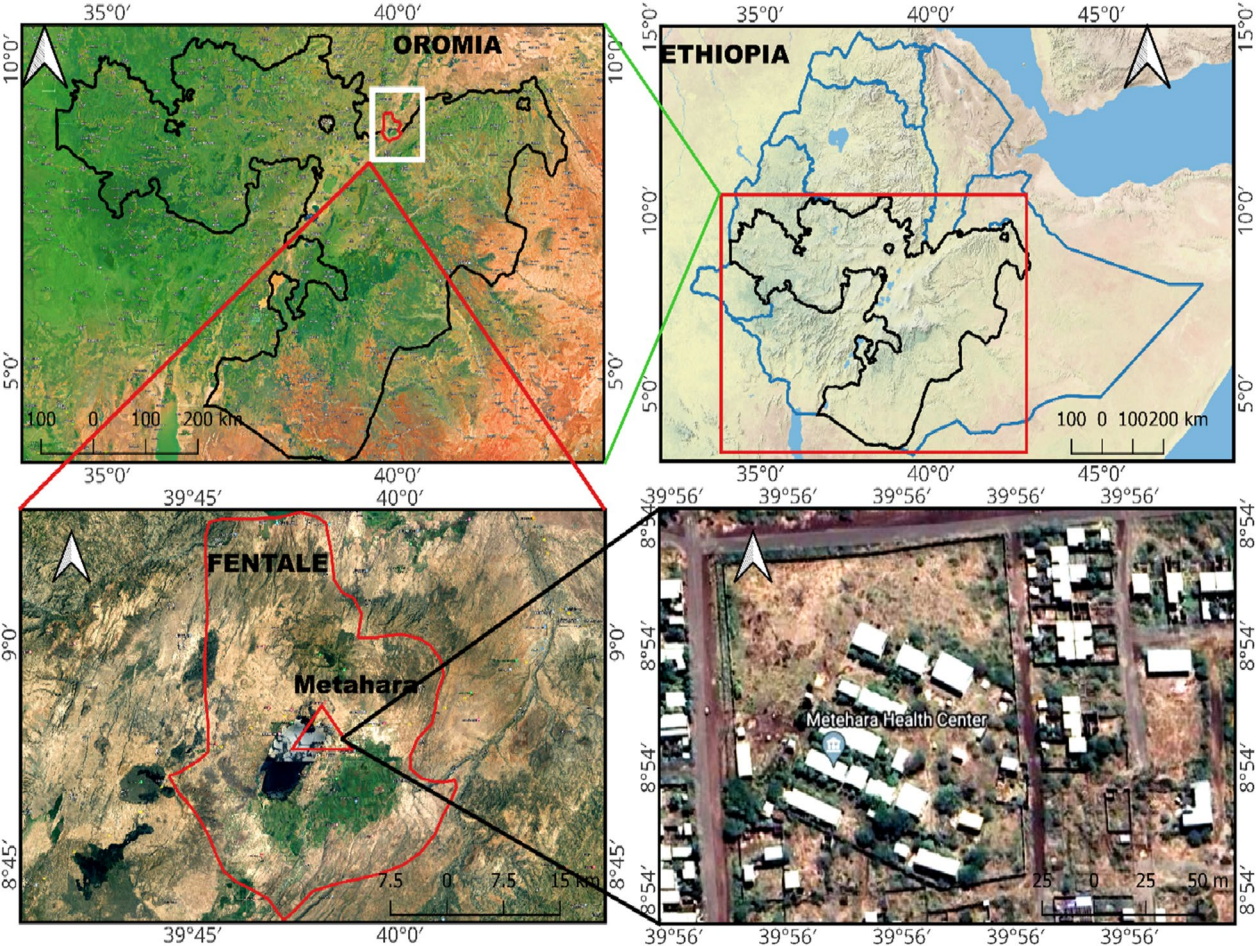
Map of study site in Ethiopia

The research was carried out at Metehara Health Centre, situated in Metehara town at coordinates 8° 86′ 95″ N and 39° 92′ 02″ E, approximately 188 km to the East of Addis Ababa (Fig. 1). Metehara hosts an estimated total population of 21,348, with 10,763 men and 10,585 women [36]. Notably, the health centre is among the public health service providers in the town and surrounding villages. Metehara experiences a hot, semi-arid climate and bimodal rainfall. The main rainy season is from July to September and a shorter season from February to April. The average annual rainfall is 571 mm. The temperature fluctuates from 24 to 34 °C year-round, with occasional drops and spikes, with the highest temperatures from May to June and the lowest from November to January [32, 37]. In the area, malaria is prevalent throughout the year, reaching peak transmission from September to December and experiencing the lowest transmission from April to May [38].

The dynamics of malaria are greatly influenced by climate variability as well as the topography of the area, which is located between Lake Beseka and the Awash River Basin [32]. Temperature fluctuations, particularly elevated temperature in May and June, promote mosquito breeding and *Plasmodium* development. Additionally, bimodal rainfall patterns provide an ideal environment for mosquito breeding during both the main and shorter rainy seasons, which may lead to an increase malaria transmission. On the other hand, the Awash River Basin and Lake Beseka provide breeding grounds for mosquitoes during the dry season. Furthermore, human activities like farming and fishing near these water bodies also create mosquito breeding sites and increase human-mosquito contact, which further influences the transmission of malaria [5, 34]. In Metehara, *P. vivax* and *P. falciparum* coexist, with *P. falciparum* predominating. Moreover, there have been reports of *An. stephensi* resistant to long-lasting insecticidal nets (LLINs) and indoor residual spraying (IRS) from a village near Metehara town [20].

The study focused on individuals suspected of malaria who had sought medical attention at Metehara Health Centre, examining blood films over the past 6 years from the 2017/18 to 2022/23 GC (2010–2015 E.C) periods.

### Study design and data source

A retrospective study based on health facility records was undertaken, utilizing the laboratory registration logbook, to analyse the six-year trend of malaria prevalence at Metehara Health Centre spanning from 2017/18 to 2022/23 G.C (2010–2015 E.C). The health centre’s malaria laboratory registration logbook served as the primary data source for this investigation. Data extracted from the logbook included information on diagnosed malaria cases, specifying months and years, identified *Plasmodium* species types, and socio-demographic details such as age and sex. The study encompassed all registered malaria blood film results, excluding only incomplete data and unreadable documents.

### Data collection

A comprehensive analysis of retrospective data spanning 6 years, from September 2017 to August 2023, was carried out to examine the trend of malaria prevalence at the Metehara Health Centre in Ethiopia. The investigation utilized the facility’s laboratory logbook, with trained laboratory personnel collecting data through the use of data extraction sheet. This sheet captured various details, including Giemsa-stained blood film results, *Plasmodium* species (such as *P. falciparum*, *P. vivax*, and mixed infections), examination specifics, and the year and month of diagnosis. In addition, socio-demographic information of the patients was recorded. The laboratory diagnosis of malaria parasite at the health centre followed the guidelines recommended by the World Health Organization (WHO).

### Data quality assurance

Initially, an evaluation was conducted to assess the comprehensiveness of the malaria registration books at the health facility, ensuring the reliability of the data. Subsequently, a dedicated data recording sheet was devised and employed to systematically capture the necessary information. Prior to data extraction, data collectors underwent thorough training on the extraction process. The investigators closely supervised the entire data extraction procedure, randomly selecting a subset of completed data collection forms and scrutinizing each for accuracy, consistency, and completeness. A final manual double check of all completed data was performed just before entry, and ultimately, the data underwent a thorough cleaning process in preparation for analysis.

### Data analysis

The gathered data were inputted into Microsoft Excel and subjected to analysis using Stata software version 14 (Stata Corporation, TX, USA). Descriptive statistics were employed to determine the frequencies and percentages of malaria prevalence across various parameters, including years, seasons, *Plasmodium* species, sex, and age. Microsoft Office Excel was utilized for the creation of descriptive figures, and the outcomes were presented through frequency distributions, tables, and graphical representations.

## Results

### Characteristics of study participants

During a six-year period from 2017/18 to 2022/23 G.C (2010–2015 E.C), a total of 59,250 Giemsa-stained malaria blood films were meticulously examined under a microscope to detect the presence of *Plasmodium* parasites. Of the participants whose blood films were examined, 62.8% were males, while 37.2% were females. Analysing the six-year trends, individuals aged between 25 and 54 years constituted the highest number of suspected and examined cases, totaling 38.4% (22,751) followed by the 15–24 age group with 31.9% (18,949) cases. The age group > 35 years old had the lowest suspected cases, accounting for 6.67% (1485) of the total (Table 1).

**Table 1:** Socio-demographic characteristics of study patients, 2017/18–2022/23

Variables	Gender % (n)		Age % (n)					
Years	Male	Female	> 5 years	5–14 years	15–24 years	25–34 years	> 35 years	Total
2017/18	69 (3328)	31 (1495)	15.9 (769)	19 (917)	25.5 (1231)	29.5 (1423)	10 (483)	4823
2018/19	63.2 (3618)	36.8 (2103)	15.5 (889)	10.7 (613)	30.3 (1732)	40.5 (2318)	3 (169)	5721
2019/20	65.4 (4915)	34.4 (2576)	13.4 (1002)	12.5 (935)	36.7 (2755)	28.5 (2134)	8.9 (665)	7491
2020/21	62.9 (5322)	37.1 (3132)	8.2 (694)	10.2 (864)	25 (2117)	51.1(4321)	5.4 (458)	8454
2021/22	64.8 (7796)	35.2 (4219)	10.5(1258)	13.9 (1674)	33 (3972)	36.5 (4388)	6 (723)	12,015
2022/23	59.1(12,281)	40.9 (8465)	8.7 (1813)	10.5 (2169)	34.4 (7142)	39.3 (8167)	7 (1455)	20,746
Total	62.8 (37,260)	37.2 (21,990)	10.8 (6425)	12.1 (7172)	31.9 (18,949)	38.4 (22,751)	6.67 (3953)	59,250

### Annual trends of malaria prevalence

**Fig. 2:**
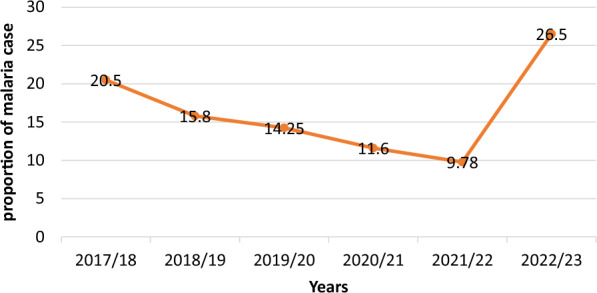
Annual trends of malaria prevalence at Metehara health centre, 2017/18–2022/23

Out of a total of 59,250 examined blood films, 17.4% (10,636) were found to be positive for *Plasmodium* species over the six-year period. The number of malaria blood film examinations at the health centre exhibited a progressive increase, with the highest number observed in 2022, closely followed by 2021. Despite an evident decline in the overall number of malaria cases over the past 6 years, dropping from 20.5% (993/4823) in 2017 to 9.8% (1176/12,015) in 2021, there was a sudden three-fold increase to 26.5% in 2022 compared to the previous year’s 9.8% (Fig. 2).

Over these 6 years, the data reveals a consistent predominance of *P. falciparum*, representing the majority 74.3% (7903/10,636) of malaria cases throughout the observed period. *Plasmodium vivax* follows as the second most prevalent species 23.8%, (2537/10,636), contributing a substantial but consistently lower proportion compared to *P. falciparum*. Additionally, mixed infections involving both *P. vivax* and *P. falciparum* exhibit a relatively minor presence 1.8% (196/10,636), contributing the smallest proportion among the *Plasmodium* species (Table 2).

**Table 2:** Prevalence and annual trends of malaria in Metehara Health Centre, 2017/18–2022/23

Year	Blood film examined	Positive % (n)	Plasmodium species		
			* P. falciparum* % (n)	* P. vivax* % (n)	Mixed* % (n)
2017/18	4823	20.5(993)	67 (665)	32.5 (323)	0.5 (5)
2018/19	5721	15.8 (907)	61.9 (562)	37.5 (341)	0.44 (4)
2019/20	7491	14.2 (1068)	76.9 (822)	22.2 (238)	0.75 (8)
2020/21	8454	11.6 (983)	61.9 (609)	36.6 (360)	1.4 (14)
2021/22	12,015	9.8 (1176)	78.9 (928)	20.4 (240)	0.68 (8)
2022/23	20,746	26.5 (5508)	78.3 (4317)	18.7 (1034)	2.85 (157)
Total	59,250	17.4 (10,636)	74.3 (7903)	23.8 (2537)	1.84 (196)

Mixed*: *P. falciparum and P. vivax*

**Fig. 3:**
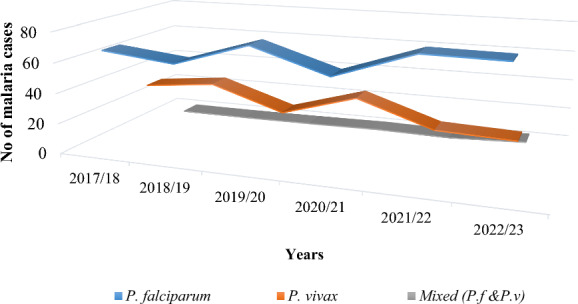
Proportion of *Plasmodium* species at Metehara health centre, 2017/18–2022/23

The trend analysis of Plasmodium species over the course of the six years showed a fluctuating trend for both species. *P. falciparum* malaria reached its peak in 2021/22, accounting for 78.9% of examined cases. In contrast, *P. vivax* was found to be highest (37.5%) in 2018/19 and the lowest (18.7%) proportion in 2022/23. Moreover, over a period of 6 years, the area experienced a fluctuating increment trend for mixed infections (*P. vivax* and *P. falciparum*), with a minimum (0.44%) cases in 2018/19 and a maximum (2.85%) in 2022/23 (Table 2). The graph visually depicts the fluctuation and composition of *Plasmodium* species, offering insights into the dynamic patterns of malaria infections at Metehara Health Centre during the specified timeframe (Fig. 3).

### Distribution of malaria with age and sex

**Fig. 4:**
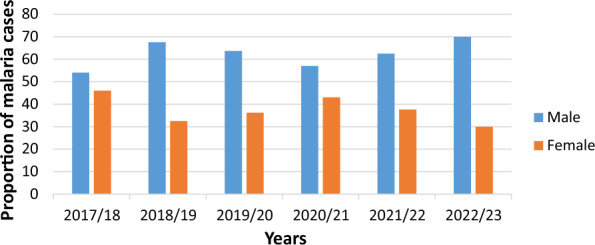
Annual trend of malaria by gender at Metehara health centre, 2017/18–2022/23

**Fig. 5:**
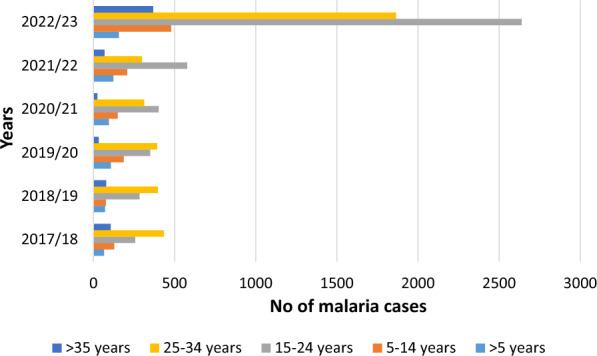
Annual trend of malaria by age group at Metehara health centre, 2017/18–2022/23

Over the six-year span from 2017/18 to 2022/23, a total of 10,636 confirmed cases of malaria were reported, with 66% (7019) occurring in males and 34% (3617) in females. Notably, males consistently exhibited a higher annual rate of malaria cases compared to females (Fig. 4) Malaria cases were observed across all age groups, with the highest percentage 42% (4509/10,636) recorded in individuals aged 15 to 24. The age group of 25 to 34 had the second-highest percentage, accounting for 34.8% (3702/10,636) of the total malaria cases (Fig. 5).

### Seasonal distribution of malaria

**Fig. 6:**
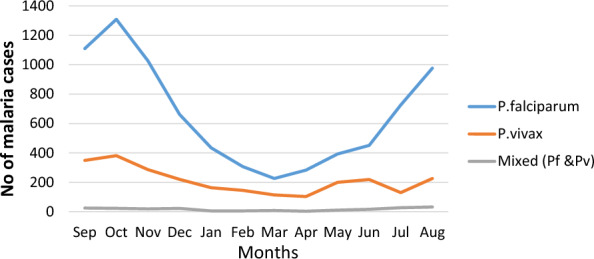
Annual trend of *Plasmodium* species by month at Metehara health centre, 2017/18–2022/23

**Fig. 7:**
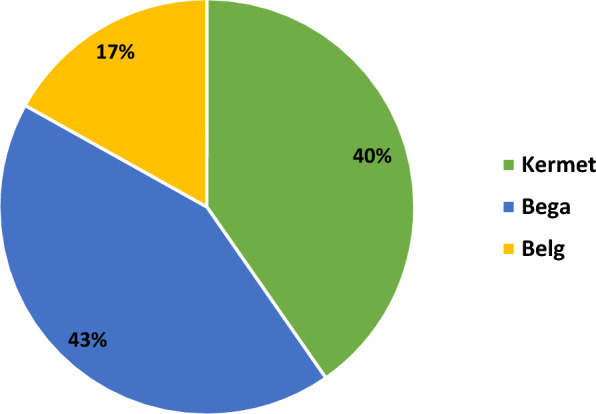
Seasonal malaria prevalence variations at Metehara health centre (2017/18–2022/23)

Despite the observable variation in total malaria trends over the 6-year study period in the examined site, malaria cases persisted consistently throughout the year. Notably, the highest peak of cases occurred in October, while the lowest number of cases was reported in March. Throughout all months, infections attributed to *P. falciparum* reached their peak (Fig. 6). Based on Ethiopian Climate Agency classification of season’s, analysis of seasonal data further revealed that the Bega season (October–January) had the highest percentage of malaria cases, accounting for 43% (4551/10,636) of the cases. About 40% (4288/10,636) of cases were reported during the Kiremt season (June to September), while the lowest 17% (1798/10,636) number of cases were reported during the Belg season (February to May) (Fig. 7).

## Discussion

According to the current study, 10,636 cases (17.4%) of malaria were microscopically confirmed over the past 6 years. Despite the implementation of various control and elimination strategies [2, 3], a substantial malaria burden persists at the study site. Comparable prevalence reports from Dembecha [39], Kaffa zone [40], Sibu Sire [41], and Dembia [42] are in line with the current study’s findings, while Mojo Health Centre [8], University of Gondar Specialized Referral Hospital [9], and Ataye [43] showed a lower prevalence in comparison. The higher prevalence observed in the present study may be attributed to the geographical features and environmental conditions of the research area. Metehara, characterized by bimodal rainfall and bordered by Awash River basin and Lake Baseka, provides an ideal breeding ground for mosquitoes. Additionally, being a crucial transportation hub connecting Adama and Awash, Metehara draws a substantial influx of travellers, drivers, and tourists, particularly during night-time, intensifying mosquito activity and consequently increasing the risk of malaria transmission.

The current study reveals a steady decline in malaria cases from 2017/18 to 2021/22, consistent with findings from other locations [8, 44, 45]. However, an unexpected threefold increase in cases in 2022/23 raises concerns about a potential malaria resurgence in the study area. The study findings emphasize the importance of vigilant monitoring and surveillance of intervention strategies to avert catastrophic events and advance national goals of zero malaria incidence and mortality by 2030 [2, 3]. Government and stakeholders have intensified efforts in malaria prevention and control to meet national strategic plan goals [3, 4]. Community initiatives, including expanded indoor residual spraying, rapid diagnosis, increased access to insecticide-treated nets, and enhanced awareness, may have contributed to the earlier decline. Conversely, disruptions caused by the COVID-19 pandemic, ongoing armed conflicts, and unrest may explain the sudden surge in malaria cases in 2022/23 [17, 24–26]. Consistent with the current findings, a different study reported a positive correlation between malaria resurgence and armed conflict. For instance, from 1991 to 2001, there was an armed conflict in Sierra Leone, which resulted in a 60% increase in malaria transmission compared to pre-conflict levels [46]. Similar to this, Côte d’Ivoire civil war of 2002–2003 led a massive increase in the prevalence of malaria and severe health system failures [47]. Furthermore, a systematic review on the causes of the malaria resurgence conducted elsewhere revealed that the interruption of vector control interventions as a result of armed conflict was the main reason for malaria increment in a number of countries, including Guinea-Bissau, Côte d’Ivoire, Guinea, Malawi, and Madagascar [48].

On the other hand climate change associated with weather events could also contribute to malaria resurgence in Ethiopia [49]. Earlier documents from malaria endemic areas revealed that malaria resurgence were frequently linked to weather events such as flooding and drought. Floods initially reduce mosquito populations by eliminating breeding sites, but as water recedes, stagnant pools form, attracting mosquitoes and increasing malaria infections [10, 50]. As an illustration the 2013 and 2014 flooding in Sudan and Uganda led to a significant increase in the malaria incidence rate compared to the previous years without floods [51–53]. Likewise, Pakistan also experienced a rise in malaria as a result of the 2010 monsoon flooding [54].

In some circumstances, drought can lead to malaria epidemics by drying up rivers and streams, allowing vector breeding, or causing water scarcity, storing water in containers which favor mosquito breeding [15, 55]. In line with this, a prolonged 2-year drought in Ethiopia’s recent history caused a peak in malaria mortality and morbidity [56]. Similarly, studies done in Venuezuela demonstrated a stronger correlation between drought and malaria mortality [57]. In addition, population immunity may have been weakened during droughts, making people more susceptible to malaria once regular rains resume [58].

This study revealed that the prevalent *Plasmodium* species identified among participating patients was *P. falciparum*, consistent with Ethiopia’s national malaria profile [3] and previous research in different regions [39, 42, 59]. However, this contrasts with earlier studies that reported a higher prevalence of *P. vivax* species [8, 9, 44]. These differences may be attributed to the severity of *P. falciparum* infection, drug resistance, and agro-climatic variations.

The investigation revealed that males were more likely than females to test positive for malaria, possibly due to their outdoor working environments involving industrial, agricultural, and day labor activities coinciding with peak mosquito biting hours. This observation aligns with studies from various malarious parts of Ethiopia [8, 9, 39, 42]. Additionally, the age group of 15–24 years exhibited the highest malaria prevalence, followed by the 25–34 age group, consistent with previous Ethiopian research [8, 9, 60]. This could be attributed to the outdoor nature of work in these age groups. In contrast, children under 5 years old showed a lower malaria prevalence, likely due to reduced mosquito exposure and the use of bed nets.

Regarding seasonal transmission dynamics, the study areas experienced year-round malaria cases, with the highest peak observed in Bega. This seasonal pattern is linked to the creation of stagnant water and higher relative humidity after the summer rainy season, providing an ideal breeding environment for mosquitoes. The seasonality observed in this study aligns with findings in Modjo [8], Dembecha [39] and Ataye [43].

### Limitation of the study

The study has limitations primarily related to the data source. Since the trend analysis relies on secondary data, the reliability of the information cannot be fully confirmed. Moreover, critical details such as participants’ clinical presentations, body temperature, treatment and diagnosis history are not available, limiting a comprehensive understanding of individual cases. The absence of information on weather conditions throughout the months, seasons, and years, as well as participants’ travel history to malarious areas, further restricts the contextual insights that could have been gained from these factors.

## Conclusion

In conclusion, the study highlights malaria as a persistent health challenge in the area, with *P. falciparum* being the predominant parasite. The Bega season (October–January) showed the highest peak of malaria cases. Notably, the disease disproportionately affects men over 15 years old, posing potential risks to the local subsistence economy. The observed 5-year decline was followed by a sharp rise, indicating a malaria resurgence. This underscores the need for a revised strategy to sustain the gains achieved by the national malaria control plan.

## Data Availability

All data generated in this study are included in the manuscript. The data sets analysed during the current study are available from the corresponding author on reasonable request.

## Abbreviations

ACT: Artemisinin-based combination therapy
EC: Ethiopian calendar
EPHI: Ethiopian Public Health Institute
FMOH: Federal Ministry of Health
G.C: Gregorian calendar
WHO: World Health Organization
